# Dual-Functionalized Electrospun PCL Membranes with Polydopamine/Gold Nanoparticles: Cytocompatibility Assessment for a Potential Cardiac Patch

**DOI:** 10.3390/polym18192408

**Published:** 2026-10-02

**Authors:** Taha Buğra Taşdelen, Busra Kilic, Özlem Eğri, Halil Murat Aydin, Sinan Eğri

**Affiliations:** 1 Graduate Education Institute, Bioengineering Division, Tokat Gaziosmanpaşa University, 60250 Tokat, Türkiye; tabutasdelen@gmail.com (T.B.T.); ozlem.egri@gop.edu.tr (Ö.E.); 2 Department of Biology, Faculty of Science, Molecular Biology Section, Hacettepe University, 06800 Ankara, Türkiye; busra2kilic@gmail.com; 3 Institute of Science, Bioengineering Division, Hacettepe University, 06800 Ankara, Türkiye; hmaydin@hacettepe.edu.tr; 4 Department of Mechanical Engineering, Faculty of Engineering and Architecture, Tokat Gaziosmanpaşa University, 60250 Tokat, Türkiye; 5 Department of Chemistry, Faculty of Arts and Sciences, Tokat Gaziosmanpaşa University, 60250 Tokat, Türkiye

**Keywords:** cardiac patches, polydopamine, electrical conductivity, human cardiac myocytes, cell compatibility

## Abstract

Myocardial infarction causes scar tissue formation and poor electrical conduction. Cardiac patches are used to functionally support damaged cardiac tissue. Electrospun polycaprolactone (PCL) membranes, one of the most investigated biocompatible and biodegradable materials to be used as cardiac patch, have limitations due to inherent hydrophobicity and weak electrical conductivity. It was aimed at developing a material in membrane form as cardiac patch to overcome these limitations for supporting damaged myocardium during therapy stage, and for better adhesion and proliferation of cardiac cells. For this purpose, the electrospun membranes were coated with polydopamine (PDA) or in situ gold nanoparticle (AuNP) decorated following PDA coating. The analyses exhibited that the PDA-AuNP modification transformed the hydrophobic PCL to a microenvironment with higher hydrophilicity and enhanced electrical conductivity with PDA concentration dependent manner. Indirect extract-based indirect cytotoxicity assessment assay on human cardiomyocyte (HCM) and human neuroblastoma cells revealed no toxicity. Seven and 14 days of HCM culture studies showed that cell adhesion and proliferation significantly increased in accordance with the increasing PDA concentration. However, highest AuNP deposition exhibited a slight decrease compared to its lower concentration group since all AuNP deposited groups exhibited increased cell adhesion and proliferation compared to their only PDA coated analogues. This decrease was attributed to extreme hydrophilicity and surface energy of the membranes diminishing the effective adhesion of the cells to fiber surface. The dual functionalized electrospun PCL membranes were concluded to be a promising candidate to be used as cardiac patch, especially the ones coated with PDA in 2 mg/mL solution and decorated with AuNPs, exhibiting an electrical conductivity enhancement by approximately 1000-fold and highest cell adhesion and proliferation.

## 1. Introduction

Acute myocardial infarction (MI) is the most fatal form of coronary artery disease, which causes more than 2.4 million fatalities annually in the United States, more than 4 million in Europe and Northern Asia, and more than one-third of mortality in nations with advanced economies [1]. Acute MI occurs when there is a substantial decrease in myocardial perfusion, resulting in cell necrosis. This process starts with acute inflammation of the infarcted area and edema formation, then progresses to mononuclear inflammation and finally to collagen scar formation [2]. The formation of collagen scar tissue, which is particularly evident in cardiovascular conditions and subsequent to MI, results in electrical insulation. The deposition of collagen leads to the formation of electrically isolated fibers in the living myocardium, thereby interrupting conduction pathways and reducing the rate of action potential propagation, ultimately contributing to the emergence of reentrant arrhythmias [3]. The information obtained supports the idea that myocardial fibrosis forms an arrhythmogenic substrate through several mechanisms, including mechanisms related to the disruption of the electrophysiological compliance of the myocardium (such as slowing action potential propagation and initiating reentry) and mechanisms related to the alteration of the electrical activity of cardiomyocytes (such as promoting depolarization and enhancing ectopic automaticity) [4]. In such cases, the resulting scar tissue needs to be removed or functionally replaced with other healthy tissues [5]. As viable alternatives to cardiac tissue transplantation for the repair of damaged myocardium, cell transplantation, left ventricular assist devices, drug therapy, and tissue engineering methods have gained prominence [6]. The myocardium’s extracellular matrix (ECM) is crucial in creating a nanofibrous environment for cardiac cell adhesion, growth, and function. As a result, polymeric structures that mimic the cardiac muscle ECM are particularly useful [7]. Cardiac patches made from synthetic or natural polymers are used to functionally support and/or treat damaged cardiac tissue [8]. The fibrous structure of electrospun scaffolds is highly beneficial for mimicking the natural ECM and providing a temporary, functional environment for cells during tissue regeneration [9]. Electrospinning is one of the most promising tissue engineering approaches currently available. This approach offers several advantages, including the ability to produce micro- to nanometer-scale fibers with a high surface area. Electrospinning has the advantages of processing a wide range of materials, a simple process, and the ability to form high-porosity fibers. As a result, interest in this field has grown, and several studies are underway to develop electrospun nanofibrous scaffolds for various tissue engineering applications [10,11]. The electrospinning method is used to produce nanofibers from a variety of materials in solution or melt form, the great majority of which are organic polymers of natural and/or synthetic origin [12]. While polyhydroxyalkanoates (PHAs) are promising bio-based alternative polyesters [13], typical PHA derivatives such as polyhydroxybutyrate (PHB) often exhibit high crystallinity and brittle mechanical behavior, making them less ideal for the highly dynamic, continuously beating environment of the myocardium [14]. In contrast, polycaprolactone (PCL), a biodegradable, semi-crystalline, aliphatic polyester, was selected as the foundational polymer in this study due to its superior elastomeric properties, highly tunable electrospinnability, and proven ability to withstand cyclic cardiac mechanical stress without premature failure [15]. PCL is a hydrophobic polymer with a glass transition temperature (Tg) of −60 °C and a melting temperature of 59 to 64 °C. PCL’s excellent solubility in various solvents, low melting temperature, compatibility when blended with other polymers, ease of processability, and biocompatibility have stimulated extensive research into its potential applications in the biomedical field [16,17].

Polydopamine (PDA) deposition approach, inspired by natural mussels, was first described by Lee and Messersmith’s group in 2007 as a simple and universal method to adjust the surface properties of a wide range of materials. PDA is a water-insoluble, strongly cross-linked polymer formed by the induced oxidation of dopamine monomer in alkaline environments [18,19]. PDA can be deposited on a variety of materials, resulting in coated surfaces with varying characteristics. PDA exhibits remarkable adhesion, metal coordination, and antioxidant properties, primarily due to catechol and primary amine functional groups. As with numerous natural polyphenols characterized by catechol structures, which serve as valuable building blocks for materials via metal chelation and diverse chemical reactions, the catechols within PDA provide efficient sites for modification [20]. Furthermore, the numerous functional groups in PDA coatings can be modified to incorporate biomolecules or medicines. As a result, PDA coatings with excellent adhesion, secondary reactivity, hydrophilicity, and biocompatibility features are used in TE applications, leaving a favorable impression for the future [21,22].

Additionally, conductive nanoparticles are often used to overcome the poor electrical conductivity of polymeric scaffolds. In this research, gold nanoparticles (AuNPs) were selected as a conductive nanocomponent rather than carbon-based alternatives such as carbon nanotubes or graphene. This interest stems from the potential of AuNPs to enhance localized electrical conductivity while avoiding the major cytotoxic effects often associated with carbon-based nanomaterials. Also, it has been suggested that incorporating AuNPs into cardiac patches enhances the synthesis of critical electrical coupling proteins, such as Connexin-43 (Cx43), in cardiomyocytes, thereby promoting coordinated contractions and improved structural organization [23,24].

Our previous work [25] focused strictly on fabrication techniques and on physicochemical, structural, and electromechanical analyses of dual-functionalized electrospun PCL membranes; the current study aims to introduce this material into a biological environment. We specifically aimed to thoroughly study the in vitro biological relationships, including indirect extract-based cytotoxicity, direct cytocompatibility, cellular metabolic activity, adhesion, and proliferation, to assess the cell-material interactions of these membranes as potential cardiac patch candidates. For this purpose, the membranes (including neat PCL, PDA-coated PCL at three varying concentrations, and in situ AuNP-doped PDA-PCL) were assessed with primary human cardiac myocytes. Moreover, human neuroblastoma cells were used not to claim a neural tissue engineering application, but instead as an additional, highly responsive human cell model to validate the overall extract-based cytotoxicity of the membranes for electrically excitable tissues.

## 2. Materials and Methods

PCL pellets (Mn = 80,000 Da) were supplied by Sigma-Aldrich (St. Louis, MO, USA) for the production of electrospun fibers. For the surface modification process, dopamine hydrochloride (99% purity) was obtained from Alfa Aesar (Kandel, Germany) to form the PDA coating. The in situ synthesis of AuNPs was carried out using gold(III) chloride (HAuCl_4_, 99.99% trace metals basis) from Sigma-Aldrich (St. Louis, MO, USA) as the metallic precursor and sodium borohydride (NaBH_4_) from Carlo Erba (Milan, Italy) as the chemical reducing agent. The experimental study comprised seven distinct groups: PCL, 0.2PDA, 2PDA, 20PDA, 0.2PDA-Au, 2PDA-Au, and 20PDA-Au.

### 2.1. Fabrication of Electrospun PCL Membranes

A 15% (*w*/*v*) polymer solution was prepared by dissolving 1.8 g PCL (80 kDa) in a solvent mixture of 10 mL chloroform and 2 mL ethanol. The mixture was continuously stirred for 16 h to ensure that the dope was totally homogeneous before electrospinning. Electrospinning was performed with a special system (Gamma High Voltage Res. Inc., Ormond Beach, FL, USA). The prepared solution was added to a 10 mL syringe fitted with a 21-gauge stainless steel needle. A programmable syringe pump was used in conjunction with a high-voltage power source and a grounded rotating mandrel to control the deposition. The electrospun membranes were fabricated under optimized conditions, such as a constant flow rate of 2 mL·h^−1^, an applied voltage of 14 kV, and a tip-to-collector distance of 10 cm. The rotating collector of 9 cm diameter was maintained at a rotation speed of 150 rpm. The experiments were conducted in a cabin at room temperature and a relative humidity between 30–40%.

### 2.2. Polydopamine Coating

The electrospun PCL membranes were modified by oxidative self-polymerization of dopamine. First, a 10 mM Tris(hydroxymethyl)aminomethane buffer was prepared in deionized water, and the pH was adjusted to 8.5 with hydrochloric acid (HCl) to create an alkaline environment conducive to polymerization. The centralized areas of the PCL membranes (2.5 × 10 cm) were cut, washed with distilled water, and dried. Then, the membranes were immersed in Tris-buffer solutions containing different concentrations of dopamine-HCl (0.2, 2, and 20 mg·mL^−1^), designated as the 0.2PDA, 2PDA, and 20PDA groups, respectively. To facilitate the formation of a stable PDA layer, the immersion process was carried out for 16 h at room temperature. After the coating process, the modified membranes were thoroughly washed with distilled water to remove any residual molecules, then allowed to air-dry.

### 2.3. In-Situ Synthesis and Deposition of AuNPs

The AuNPs were deposited on the surfaces of the functionalized membranes by a two-stage in situ reduction method. A precursor solution in water was prepared by mixing gold(III) chloride trihydrate (HAuCl_4_) at 0.2 mM and adjusting the pH to 4.0. The dried PCL membranes with PDA coating (0.2, 2, and 20 mg·mL^−1^ coating groups) were immersed in the metallic precursor solution at room temperature for 1 h. In this method, the catechol and amine groups in the PDA layer quickly bonded to the Au^3+^ ions. After the ion-adsorption step, the membranes were immediately transferred to a new ice-cold solution of 2 mM sodium borohydride (NaBH_4_). This reduction process was allowed to continue for 5 min, during which the adsorbed gold ions were chemically transformed into metallic nanoparticles directly on the fiber surfaces. The composites obtained were identified as 0.2PDA-Au, 2PDA-Au, and 20PDA-Au. The obtained composites were collected, thoroughly washed with deionized water to remove residual chemicals, and then dried in ambient air.

### 2.4. Scanning Electron Microscopy (SEM)

Circular specimens with a diameter of 6 mm were removed from the matrices to evaluate surface morphology. After that, these samples were vacuum-sputter-coated with gold for ten minutes (Quorum Q150RES, Birmingham, UK) to ensure electrical conductivity. Then, the matrices were analyzed by scanning electron microscopy (SEM; Tescan Mira3 XMU, Brno, Czech Republic) at different magnifications to evaluate their structural features.

### 2.5. Density and Porosity Assessment

Gravimetric techniques were used to assess the membranes’ physical properties. The density (ρ) and average specific volume (cm^3^·g^−1^) of square specimens (1 × 1) were precisely measured by weight. Equation (1) was then used to determine the porosity percentage:

Porosity (%) is equal to [(V_m_ − V_0_)/V_m_] × 100 (1)

where V_0_ is the specific volume of the pure polymeric film, and V_m_ is the specific volume of the electrospun membranes.

### 2.6. Surface Wetting Characteristics

In accordance with ASTM D7334-2013 [26], static water contact angle (WCA) measurements were performed to assess the hydrophilicity of the membranes using an Attension Biolin Scientific system (Attension, Biolin Scientific AB, Gothenburg, Sweden). Each specimen had a 4 μL droplet of deionized water applied to its surface. To evaluate the wetting behavior of the composites, contact angles for each group were measured in triplicate, and the average contact angles were computed from the digital images.

### 2.7. Water Uptake (Swelling)

The 6 mm circular matrices were submerged in phosphate-buffered saline (PBS) to assess the membranes’ ability to absorb liquid. Before weighing, extra surface water was carefully removed from the samples, which were taken out at predetermined intervals (3, 6, 9, 12, 15, 30, 60, 90, and 120 min). Equation (2) was used to calculate the water uptake ratio;

Swelling Ratio (%) = [(W_i_ − W_0_)/W_0_] × 100 (2)

where W_0_ and W_i_ stand for the initial dry weight and the weight at time t, respectively.

### 2.8. Electrical Conductivity Measurements

The four-probe method was used to measure the electrical conductivity (σ) of the composite membranes. The sheet resistance was measured in triplicate for each group. Equation (3) was used to calculate the conductivity values;

σ = [ln2/(πt)] × (I/V)(3)

where t is the thickness of the membrane, V is the measured voltage, and I is the applied current.

### 2.9. Tensile (Mechanical) Tests

To evaluate the mechanical properties of the fabricated electrospun matrices, tensile testing was conducted in accordance with EN ISO 13934-1:2013 [27] using a universal testing machine (Autograph AGS-X, Shimadzu, Kyoto, Japan). Specimens measuring 0.5 cm × 10 cm were mounted between the grips, and testing was performed at room temperature at a constant crosshead speed of 50 mm·min^−1^. Stress–strain curves were recorded for each sample to determine the ultimate tensile strength (UTS), strain at break, and elastic modulus. Five replicates were tested per group, and mean values were reported alongside standard deviations.

### 2.10. In Vitro Cytocompatibility of Electrospun Membranes

#### 2.10.1. Extract-Based Indirect Cytotoxicity Assessment

The in vitro cytotoxicity of PCL, PDA-coated, and PDA-coated-Au-deposited electrospun membranes was evaluated using an indirect MTT assay. Prior to extraction, the membranes were sterilized with 70% (*v*/*v*) ethanol for 2 h, washed three times with PBS, and exposed to UV light for 20 min on each surface. Sterilized membranes were incubated in the respective cell culture media at an extraction ratio of 0.1 g·mL^−1^ at 37 °C for 24 h to obtain membrane extracts. Human neuroblastoma SH-SY5Y cells (ATCC CRL-2266, Manassas, VA, USA) were cultured in DMEM/F-12 Medium (Gibco, Waltham, MA, USA), while primary human cardiac myocytes (HCM; Cat. No. C-12810, PromoCell, Heidelberg, Germany) were cultured in complete Myocyte Growth Medium (Cat. No. C-22070, PromoCell, Heidelberg, Germany), prepared according to the manufacturer’s instructions by aseptically adding the supplied SupplementMix to the basal medium. The final supplemented medium contained 5% fetal calf serum, 0.5 ng·mL^−1^ recombinant human epidermal growth factor, 2 ng·mL^−1^ recombinant human basic fibroblast growth factor, and 5 µg·mL^−1^ recombinant human insulin. HCM at passages 6–8 were maintained at 37 °C in a humidified atmosphere containing 5% CO_2_, and the culture medium was replaced every 2 days. Both cell types were seeded in 96-well plates at a density of 1 × 10^4^ cells/well and incubated for 24 h. The cells were then exposed to the respective membrane extracts for 24 and 48 h. At each time point, MTT solution (5 mg mL^−1^) was added and incubated for 3 h at 37 °C. The resulting formazan crystals were dissolved in dimethyl sulfoxide (DMSO, Sigma-Aldrich, St. Louis, MO, USA), and absorbance was measured at 570 nm using a microplate reader (BioTek, Winooski, VT, USA). Cell viability was expressed as a percentage of the untreated control.

#### 2.10.2. Direct Cytocompatibility and Metabolic Activity Assessment Assay

Long-term cell proliferation and metabolic activity on PCL, PDA-coated, and PDA-coated-Au-deposited electrospun membranes were evaluated using the Alamar Blue assay. Primary HCM were seeded directly onto the sterilized membranes at a density of 3 × 10^4^ cells/membrane and cultured at 37 °C and 5% CO_2_ for up to 14 days. On days 7 and 14, the cell-seeded membranes were carefully transferred to separate fresh wells prior to the Alamar Blue assay. Alamar Blue solution was then added to the transferred membranes, which were incubated for 4 h. Following incubation, 200 µL of the solution was transferred to a 96-well plate, and absorbance was measured at 570 nm with a reference wavelength of 600 nm using a microplate reader (BioTek, Winooski, VT, USA).

#### 2.10.3. Live/Dead Cell Staining

Cell viability on PCL, PDA-coated, and PDA-coated-Au-deposited electrospun membranes was evaluated using a 30002-T BIOTIUM Viability/Cytotoxicity Assay Kit (Biotium, Freemont, CA, USA) for Animal Live and Dead Cells according to the manufacturer’s instructions. Primary HCM (3 × 10^4^ cells/membrane) were seeded directly onto the sterilized membranes and subsequently stained with calcein-AM (2 μM) and ethidium homodimer-III (4 μM) for 30 min at room temperature. Live/dead cell staining was performed on days 7 and 14 after cell seeding. Live and dead cells were visualized using a fluorescence microscope (Leica DM IL LED, Wetzlar, Germany).

### 2.11. Statistical Analyses

The data were statistically analyzed using the one-way ANOVA variance analysis method (IBM SPSS Statistics 27, IBM Corp., Armonk, NY, USA) and/or Prism 8 (GraphPad Software Inc., San Diego, CA, USA), followed by post hoc multiple tests (Tukey, Bonferroni). Significance threshold was set to *p* < 0.05 (*). Other significance levels used: *p* < 0.01 (**), *p* < 0.001 (***), and *p* < 0.0001 (****).

## 3. Results

### 3.1. SEM Analysis

**Figure 1:**
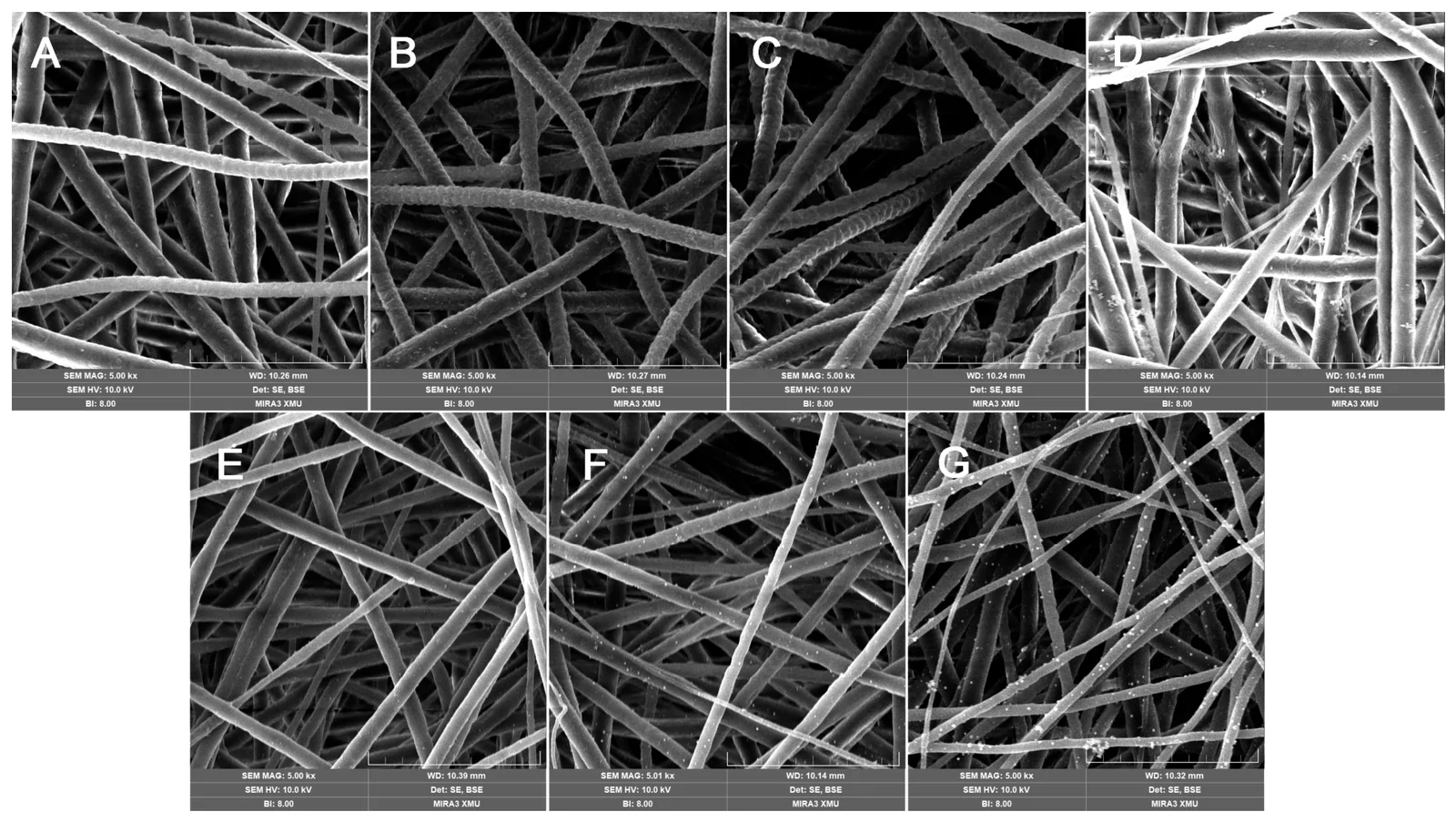
SEM Images of Non-Coated, PDA-Coated, and AuNP-Loaded PCL fibers. SEM micrographs of (**A**) non-coated electrospun PCL; (**B**–**D**) PCL fibers coated with 0.2, 2, and 20PDA, respectively; (**E**–**G**) AuNP-loaded PCL fibers prepared with 0.2 mM Au precursor and 0.2, 2, and 20PDA, respectively; (scale bar = 20 µm).

Morphological modification of electrospun PCL fibers after PDA coating and subsequent AuNP loading was observed by SEM (Figure 1), and fiber- and AuNP- diameters calculated using ImageJ (1.54g) are summarized in Table 1.

Electrospun fibers of neat PCL (Figure 1A) had a bead-free, smooth, and uniform surface morphology. When the surfaces of the PCL fibers were coated with PDA at concentrations of 0.2, 2, and 20PDA (Figure 1B, Figure 1C and Figure 1D, respectively), the structural integrity of the microfiber network was maintained; however, substantial changes in surface morphology and fiber diameters were observed.

Image analysis of the SEM images revealed that as the PDA concentration increased, the deposited layer on the polymer surface thickened, resulting in larger fiber diameters. ImageJ analysis revealed that neat PCL fibers had an average diameter of 2.44 ± 0.52 µm. After coating, this value increased to 2.49 ± 0.38 µm for 0.2PDA, 2.62 ± 0.17 µm for 2PDA, and 2.91 ± 0.31 µm at the highest concentration of 20PDA. The concentration-dependent thickening trend and increased surface roughness are measurable markers of successful PDA surface modification. The effect of surface changes on the diameter distribution of electrospun fibers was determined using one-way ANOVA. The study found no statistically significant variations in fiber diameters between the experimental groups (*p* = 0.630). Minor variations in average fiber diameters observed following PDA coating and AuNP deposition, ranging from 2.44 ± 0.52 µm for pure PCL to 2.91 ± 0.31 µm for 20PDA, did not reach statistical significance according to Tukey’s HSD and Bonferroni post-hoc multiple comparison tests (*p* > 0.05). Furthermore, grouping all experimental sets into a single subset in the homogeneous subsets analysis confirms that the surface modification procedures preserved the baseline morphological dimensions and structural architecture of the PCL fibrous membrane, without causing structural degradation.

The PDA-coated PCL fibers provided a functional redox platform for the chemical synthesis and surface immobilization of AuNPs. In the high-magnification SEM images of the AuNP-loaded samples prepared with a constant gold precursor concentration of 0.2 mM (Figure 1E–G), AuNPs firmly attached to the fiber surfaces are clearly distinguished as bright dots. A similar PDA-dependent thickening trend in fiber diameters was also observed in the AuNP-loaded groups; the average fiber diameters for the 0.2, 2, and 20PDA-Au samples were recorded as 2.48 ± 0.27 µm, 2.64 ± 0.32 µm, and 2.74 ± 0.29 µm, respectively.

Analysis of the images showed that the surface density of AuNPs was directly related to the initial PDA concentration. In the lowest PDA concentration (02PDA, Figure 1E), the AuNPs on the fiber surface were not very dense. However, at a concentration of 2PDA (Figure 1F) and 20PDA (Figure 1G), the density of particles covering the fibers increased dramatically. The catechol and amine groups of PDA serve as robust anchoring sites to bind gold ions (Au^3+^) onto the fiber surface, while NaBH_4_ acts as the primary reducing agent to convert them into metallic gold (Au^0^) [28]. The size distribution measurement of the AuNPs synthesized on the surface exhibited a broad range. The average particle diameter for the 20PDA-Au group was 88 ± 31 nm. The minimum and maximum particle diameters were 41 nm and 134 nm, respectively.

### 3.2. Density and Porosity

**Figure 2:**
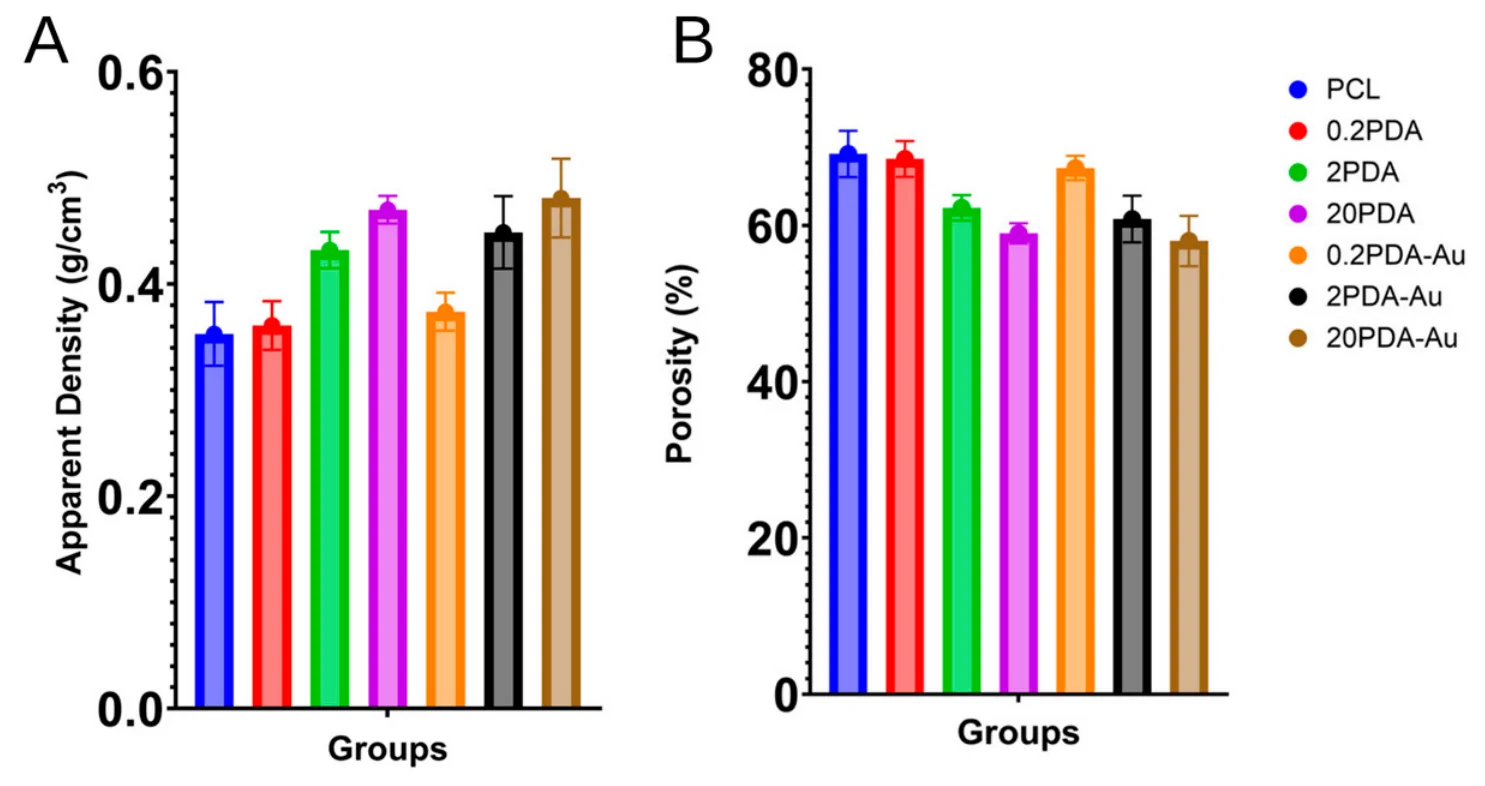
Apparent density (**A**) and porosity (%) (**B**) of the neat and PDA-Au modified membranes.

Both apparent density and porosity values for all experimental groups were determined and summarized in Figure 2 and Table 2. In the apparent density tests, the mean density of the control group (neat PCL membranes) was approximately 0.35 g·cm^−3^. Coating with PDA led to a concentration-dependent increase in matrix mass. The density of the 0.2PDA group (0.36 g·cm^−3^) was not significantly different from the neat PCL, while the density of the 2PDA and 20PDA groups increased to 0.43 g·cm^−3^ and 0.47 g·cm^−3^, respectively. The in situ reduction in AuNPs maintained this overall upward trend. The highest overall density, about 0.48 g·cm^−3^, was observed in the 20PDA-Au membrane.

Porosity measurements, on the contrary, showed a gradual reduction profile in parallel with the increase in material density. The neat PCL membranes exhibited the highest porosity, about 69%. The low-concentration modification procedure groups, 0.2PDA (approximately 68%) and 0.2PDA-Au (approximately 69%), showed no significant decrease (*p* > 0.05) compared with the control group, and porosity was mostly maintained. However, the inter-fiber void volumes decreased with increasing coating concentration, with 2PDA and 2PDA-Au groups measured at around 62% and 66%, respectively. The most intensive physical and chemical modifications were applied to the 20PDA and 20PDA-Au groups, which exhibited the lowest porosity values of 59% and 58%, respectively (*p* < 0.001).

### 3.3. Surface Wetting Characteristics

**Figure 3:**
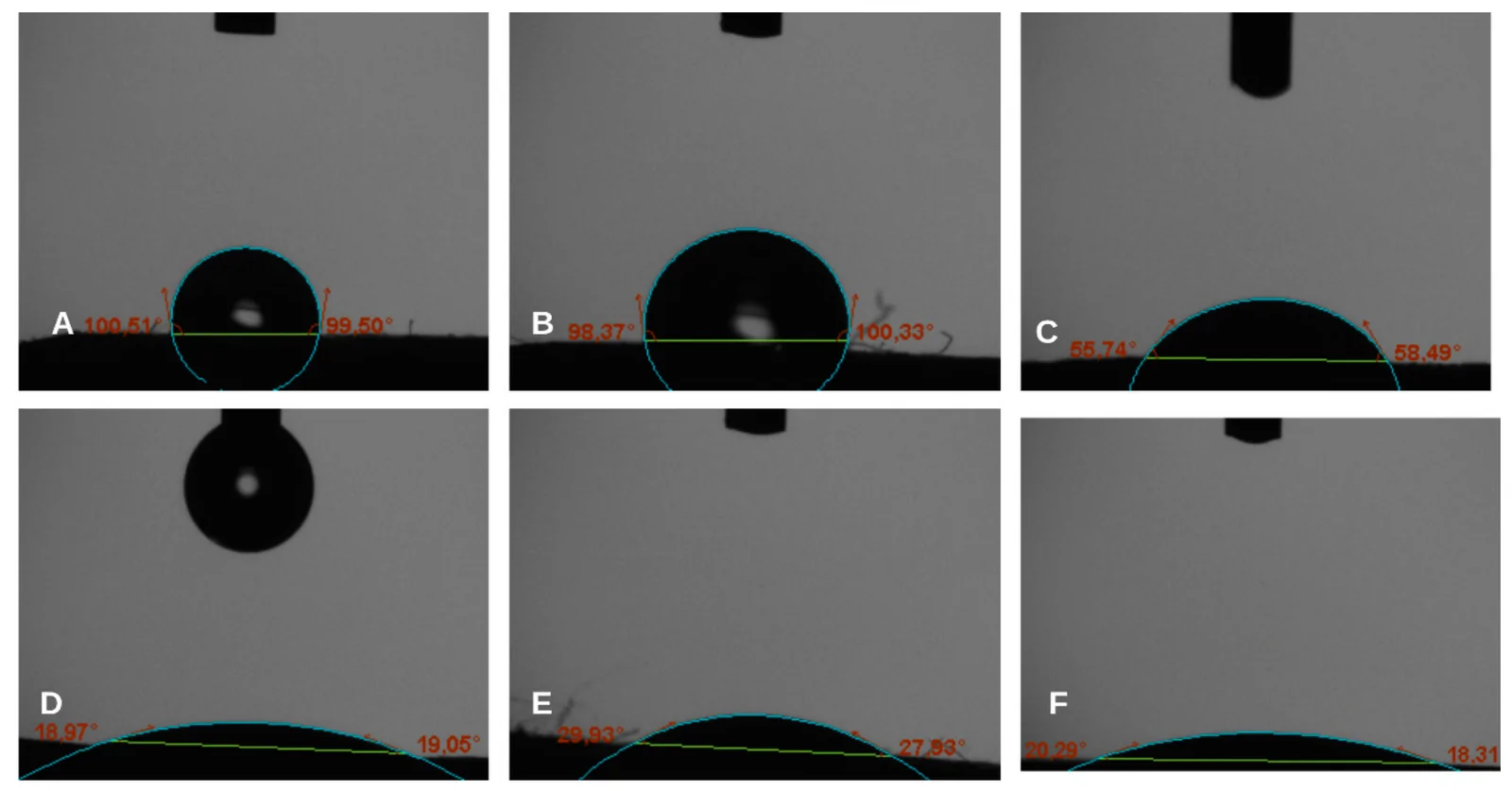
Representative water contact angle images of the test groups; (**A**) PCL, (**B**) 0.2PDA, (**C**) 2PDA, (**D**) 20PDA, (**E**) 0.2PDA-Au, (**F**) 2PDA-Au.

The surface wettability and interfacial wetting profiles of the fabricated matrices were evaluated by measuring the static water contact angle across all experimental groups, and the results are summarized in Table 2. Representative images of the contact angle measurements are presented in Figure 3. Neat PCL electrospun membrane, utilized as a control, showed an average contact angle of 100.05 ± 0.254° (Figure 3A), confirming its typical hydrophobic character [29].

The PDA coating on the fibrous membranes decreased their contact angles, which varied with concentration. For the group functionalized with the lowest PDA concentration (02PDA), the contact angle was measured at 97.24° ± 4.750 (Figure 3B), indicating that this initial treatment did not significantly alter surface wettability (*p* > 0.05). On the other hand, when the monomer solution concentration was increased to 2PDA, a significant reduction in the contact angle to 57.42° ± 1.907 (Figure 3C) was observed (*p* < 0.001), indicating that the surface profile shifted to a hydrophilic state. At the highest coating concentration of 20PDA, the contact angle decreased to the lowest value of 16.57°± 0.510 (Figure 3D) (*p* < 0.001), indicating enhanced hydrophilicity.

Additionally, a further decrease in the wetting angles of the modified membranes was observed after the subsequent addition of 0.2 mM HAuCl_4_ precursor solution for the in situ reduction and immobilization of AuNPs. The 0.2PDA-Au group showed a sharp decrease in contact angle to 27.30° ± 0.87 (Figure 3E). This value is significantly lower than that of both neat PCL and its AuNP-free counterpart (the 0.2PDA group). Furthermore, the 2PDA-Au group showed a greater improvement in wettability with an average contact angle of 18.30° ± 1.79 (Figure 3F).

In contrast, the 20PDA-Au membrane could not be measured to yield a stable, reproducible static contact angle. When the droplets were poured onto this particular membrane, the liquid immediately lost its spherical boundary, spread rapidly across the interface within a few tenths of a second, and was fully imbibed by the internal fibrous network by capillarity.

### 3.4. Water Uptake (Swelling)

**Figure 4:**
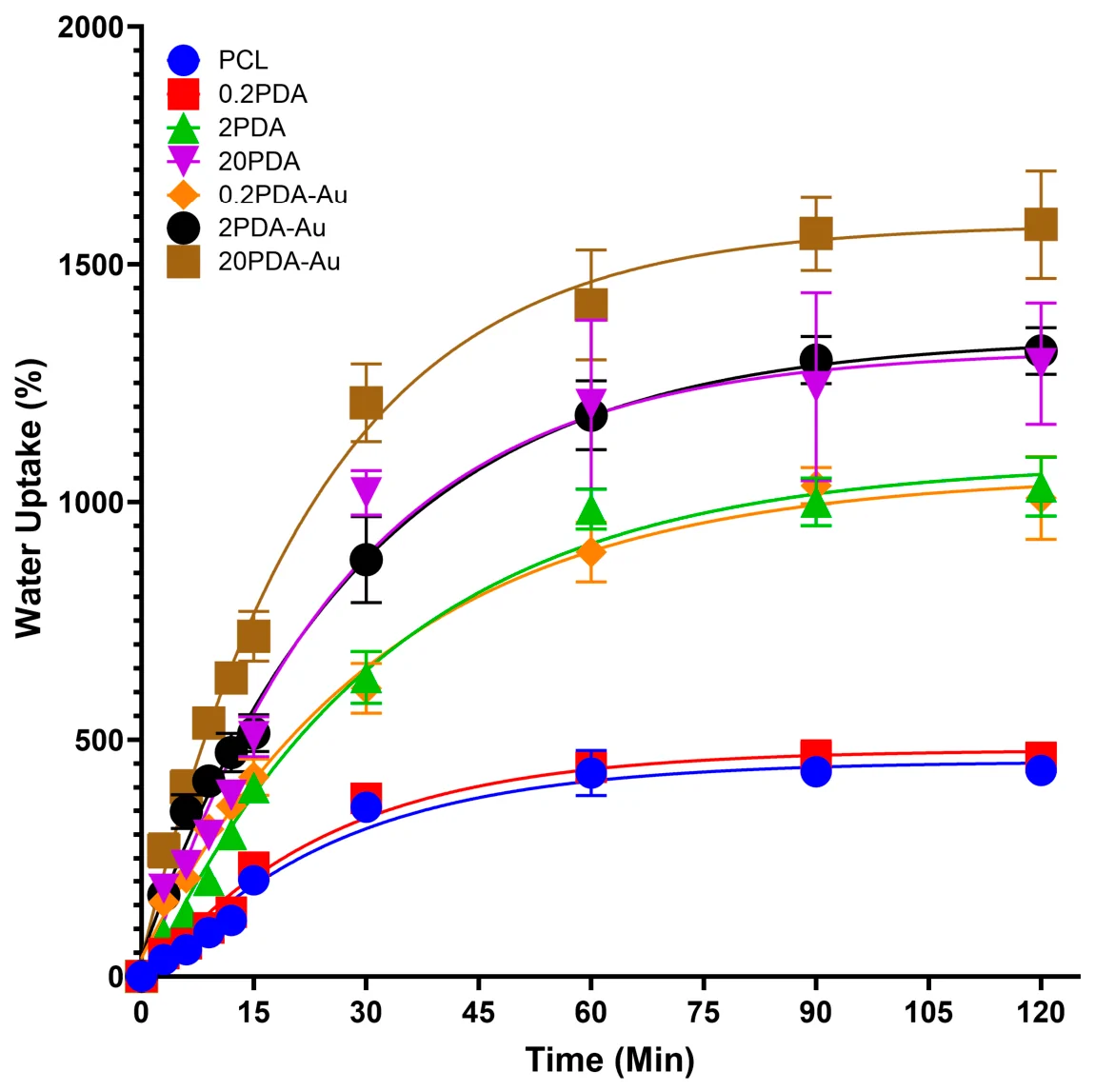
Water uptake profiles of the test groups over a 120-min period.

The time-dependent swelling tests were carried out for 120 min (Figure 4) to determine the liquid absorption capacities and the water uptake kinetics of the fabricated membranes. The neat PCL electrospun membranes exhibited the lowest liquid absorption profile, with a balanced water uptake ratio of approximately 435% at the end of the 120 min evaluation.

Surface functionalization with PDA led to a concentration-dependent increase in the membranes’ swelling capacities. The membranes coated with the lowest concentration (0.2PDA) exhibited a swelling profile very close to that of the neat PCL control group, resulting in a final water uptake of about 460%. However, the liquid absorption capacity increased significantly with increasing PDA concentration (*p* < 0.001). The maximum water uptake ratios for the 2PDA and 20PDA groups reached approximately 1032% and 1290%, respectively, at 120 min.

The in situ reduction and immobilization of AuNPs on the PDA-deposited fibers further enhanced the water uptake capacities of all corresponding groups. The 0.2PDA-Au membranes exhibited a water uptake ratio of nearly 1000%, which is significantly higher than that of the AuNP-free membranes (*p* < 0.001). Similarly, the 2PDA-Au groups showed a higher final water uptake ratio of approximately 1300%. The highest liquid absorption was seen in the 20PDA-Au group. This specific membrane exhibited rapid initial water uptake, exceeding 1200% in the first 30 min, and ultimately achieved the best overall swelling capacity, reaching a balanced ratio of around 1580% at 120 min.

### 3.5. Mechanical Properties

The mechanical properties of the fabricated electrospun fibers were determined with tensile tests for neat-, PDA modified-, and PDA-AuNP modified- PCL membranes. Analyses were performed on ultimate tensile strength (UTS), fracture strain, and modulus of elasticity (Young’s modulus) for each group, and the results were summarized in Table 3 (representative stress–strain curves are presented in Figure S1).

The elastic modulus of 41.71 ± 5.63 MPa, a fracture strain of 4.68 ± 1.51 mm·mm^−1^, and an average UTS of 7.42 ± 1.60 MPa were all observed for the neat PCL electrospun membranes. The fiber surface modification with PDA at different concentrations (0.2, 2, and 20PDA) and decoration with AuNPs resulted in slight changes in the mean values of mechanical properties. Increasing PDA concentration to 20PDA resulted in slightly higher mean tensile strength (8.55 ± 1.87 MPa for 20PDA) and elastic modulus (46.32 ± 7.09 MPa for 20PDA and 47.65 ± 7.46 MPa for 20PDA-Au), as well as a tiny reduction in fracture strain. However, one-way ANOVA revealed no significant differences among the groups for any of the characteristics analyzed (*p* > 0.05). These findings indicate that surface modification with PDA and in situ decoration with AuNPs did not result in structural degradation or embrittlement, thereby preserving the overall mechanical integrity and flexibility of the PCL nanofibrous membranes.

The mechanical properties of the fabricated membranes were compared with those of natural myocardium to determine their potential for cardiac applications. The native human heart muscle is soft and highly elastic, with a modulus of elasticity in the range of 0.02–0.5 MPa and UTS of 0.1–0.5 MPa. It is suggested that a cardiac patch should have higher mechanical strength than a normal heart to withstand the stresses of the heart’s contraction and relaxation phases [30,31]. The mechanical strength of the electrospun membranes was far beyond these values.

These values are higher than the physiological mechanical ranges of native heart tissue; this mechanical profile can be very favorable for specific cardiac treatments. MI severely weakens the ventricular wall. In this case, cardiac patches made from synthetic polymers, especially relatively stiff polymers such as PCL, are often used to provide temporary structural support and crucial mechanical reinforcement of the infarcted area [32]. Furthermore, fabricated fibers exhibit high fracture strain, indicating high flexibility. The PDA-PCL-Au membranes fabricated are therefore stiffer than the native myocardium but provide a durable, flexible, and structurally sound environment, making them highly appropriate as reinforcing cardiac patches to minimize unfavorable ventricular remodeling.

### 3.6. Electrical Conductivity

The electrical characteristics of the fabricated membranes were evaluated by measuring surface electrical conductivity (σ) using four probe tests, and the results are summarized in Table 4. As expected from its insulating polymeric nature, the neat PCL membrane was the control group, showing the lowest conductivity profile of 3.01 × 10^−8^ ± 2.60 × 10^−9^ S·cm^−1^. PDA coating on fiber surfaces enhanced the electrical conductivity values. The electrical conductivity was increased to 5.87 × 10^−7^ ± 1.42 × 10^−7^ in the 0.2PDA and 2PDA groups 5.53 × 10^−7^ ± 1.82 × 10^−7^ S·cm^−1^. The 20PDA group exhibited a further increase at the highest coating concentration with an electrical conductivity of 8.96 × 10^−6^ ± 6.60 × 10^−6^ S·cm^−1^.

The in situ reduction in AuNPs on the PDA coated fibers provided a significant enhancement of the surface electrical conductivity in all the measured groups (*p* < 0.05). The conductivity of the 0.2PDA-Au membrane was 1.16 × 10^−6^ ± 3.03 × 10^−7^ S·cm^−1^. The increase in the concentration of the underlying PDA significantly enhanced the effect, reaching 1.77 × 10^−5^ ± 3.78 × 10^−7^ S·cm^−1^ for the 2PDA-Au group. The PDA-Au group (20PDA-Au) had the greatest surface conductivity with the maximum value of 5.85 × 10^−5^ ± 2.38 × 10^−5^ S·cm^−1^.

### 3.7. In Vitro Cytocompatibility of Electrospun Membranes

**Figure 5:**
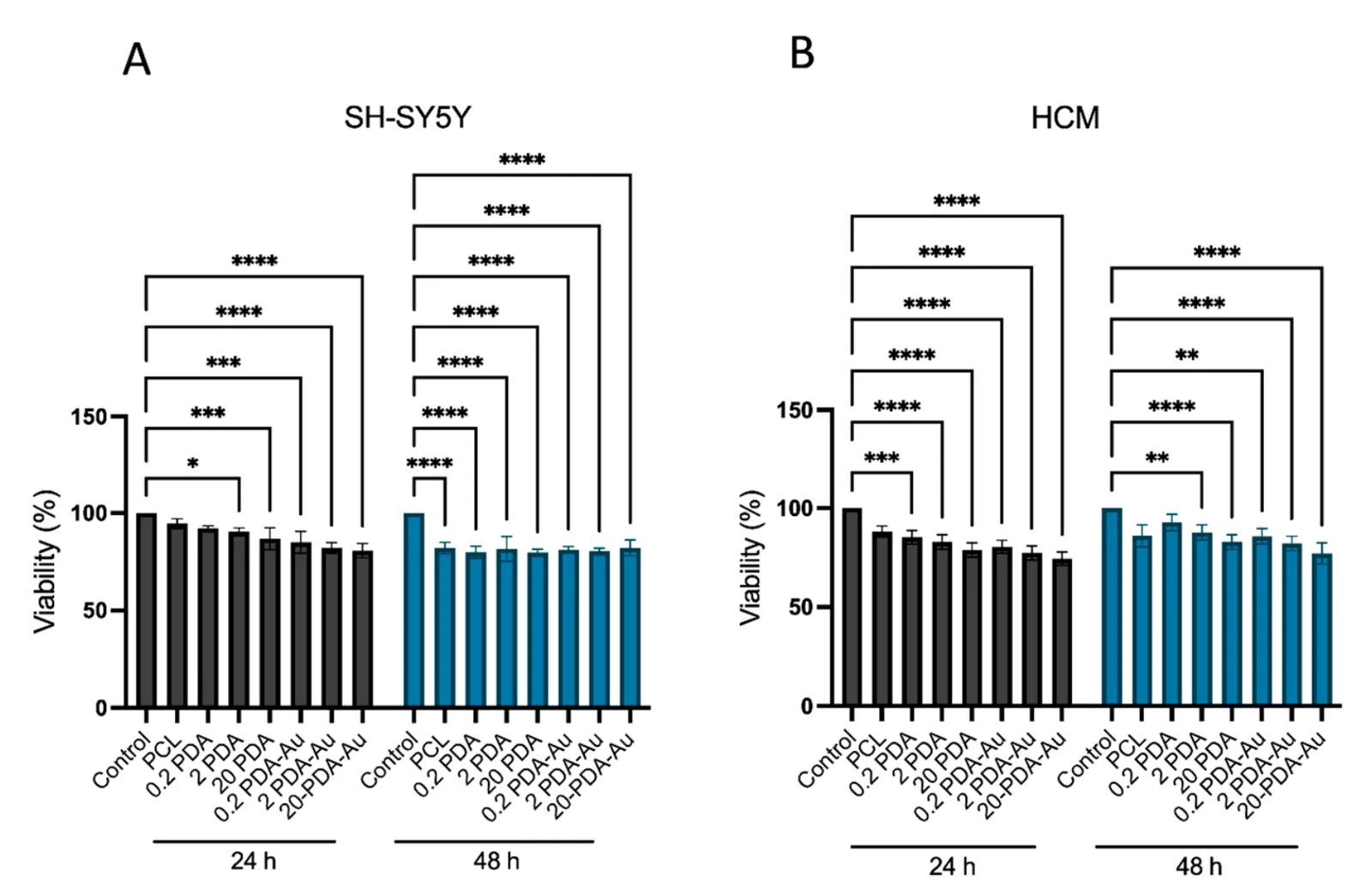
Cytotoxicity evaluation of the PCL and PDA/Au modified samples on (**A**) SH-SY5Y and (**B**) HCM cell lines over 24 and 48 h. Two-way ANOVA was performed to compare multiple groups, and the Tukey post hoc test was applied. n: 3, *p* > 0.05 (ns), *: *p* < 0.05; **: *p* < 0.01; ***: *p* < 0.001; ****: *p* < 0.0001. Control: unstimulated group, PDA: PDA coated groups, and PDA-Au: AuNPs decorated PDA coated groups.

The in vitro cytotoxicity of the fabricated fibrous membranes (PCL, 0.2PDA, 2PDA, 20PDA, 0.2PDA-Au, 2PDA-Au, and 20PDA-Au) was evaluated by extract-based indirect cytotoxicity assessment assay using SH-SY5Y neuroblastoma and primary HCM cell lines at 24 and 48 h of incubation (Figure 5A,B). After 24 h of exposure, neat PCL membranes exhibited a cell viability of 94.55%, while a slight concentration-dependent decrease was observed as the PDA coating concentration increased (92.23% for 0.2PDA, 90.26% for 2PDA, and 86.83% for 20PDA). Following AuNP incorporation, cell viability was 84.93%, 82.13%, and 80.71% for the 0.2PDA-Au, 2PDA-Au, and 20PDA-Au groups, respectively. Statistical analyses revealed that the viability values of the modified groups were significantly lower than the control group (*p* < 0.05, *p* < 0.001, *p* < 0.0001). The cell viability of SH-SY5Y after 48 h of incubation was stable between 79.72% and 82.11% for all membrane extracts, including neat PCL, and was statistically significantly different (*p* < 0.0001) compared to the control group (Figure 5A).

The cell viability of 88.10% in the pure PCL group was measured at the end of 24 h in assays conducted on primary HCM cells, the primary target cell type for cardiac tissue engineering applications. A minor reduction in viability rates was recorded for PDA coating and AuNP modifications, showing a similar trend to SH-SY5Y cells, while the viability was recorded as 74.49% in the 20PDA-Au group, where the most intense chemical modification was performed (*p* < 0.0001 compared to the control). After 48 h, cell viability increased to 92.75% in the 0.2PDA group. Viability rates in all other modified groups (including those containing AuNPs) after 48 h ranged from 77.07% to 87.69%, with statistically significant differences (*p* < 0.001, *p* < 0.0001) compared to the control group (Figure 5B).

Consequently, the PDA coating and the incorporation of AuNPs resulted in significant decreases in cell viability compared to the unstimulated group (control) (Figure 5A,B), while cell viability remained above 70% in all tested groups. The lowest viability value (74.49%) observed across all cell lines, time points, and modification groups tested is still higher than the toxicity threshold (70% cell viability). Overall, the extract-based indirect cytotoxicity assessment results suggest that the PDA- and AuNP-modified PCL membranes exhibited limited cytotoxicity and maintained cell viability under the tested conditions.

**Figure 6:**
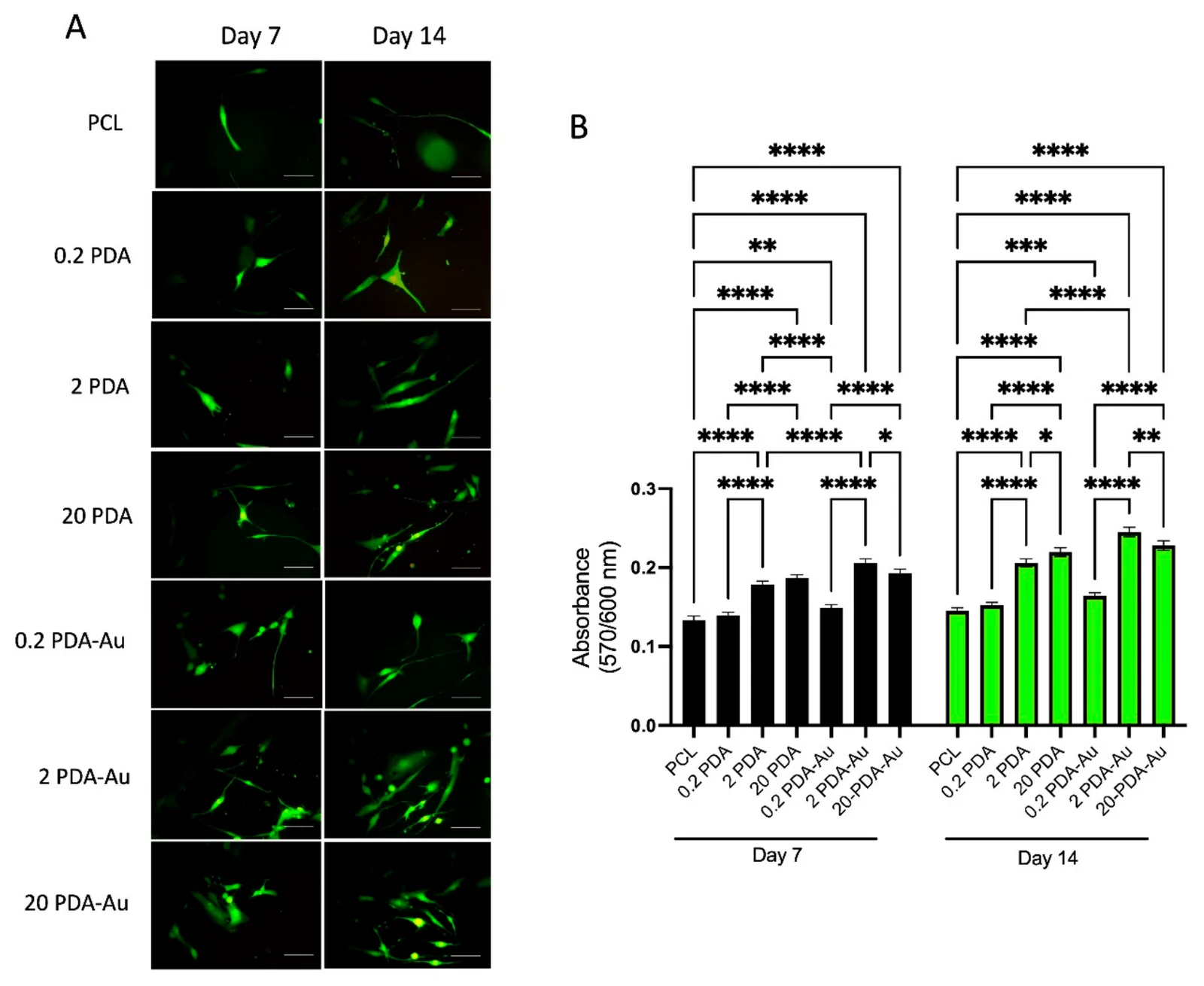
Assessment of long-term HCM cell behavior on fibrous scaffolds. (**A**) Fluorescence microscopy images and (**B**) Direct cytocompatibility and metabolic activity assessment results on days 7 and 14. Two-way ANOVA was performed to compare multiple groups, and the Tukey post hoc test was applied. n: 3, *p* > 0.05 (ns), * *p* < 0.05; ** *p* < 0.01; *** *p* < 0.001; **** *p* < 0.0001. PDA: PDA coated groups, and PDA-Au: AuNPs decorated PDA coated groups.

The long-term proliferation and metabolic activity of primary HCM on the fabricated fibrous membranes (PCL, 0.2PDA, 2PDA, 20PDA, 0.2PDA-Au, 2PDA-Au, and 20PDA-Au) were evaluated on days 7 and 14 using the direct cytocompatibility and metabolic activity assessment assay and EthD-III/calcein-AM fluorescence staining (Figure 6).

On day 7, neat PCL membranes (hydrophobic) exhibited the lowest metabolic activity. Metabolic activity was significantly enhanced by PDA modification in a concentration-dependent manner. Cell activity in the 0.2PDA group was similar to that of PCL, whereas higher metabolic activity was observed in the 2PDA and 20PDA groups (*p* < 0.0001). AuNP incorporation further increased metabolic activity, with the highest values observed in the 2PDA-Au followed by the 20PDA-Au group, where the AuNP-decorated group exhibited a significant difference with its PDA coated only form (2PDA-Au and 2PDA) (*p* < 0.0001), while 20PDA-Au did not show any significance with its PDA coated only form (*p* > 0.05) (Figure 6B).

On day 14, absorbance values increased across all membrane groups compared with day 7, indicating a time-dependent increase in HCM metabolic activity. Similar to the 7-day results, neat PCL and 0.2PDA exhibited the lowest values, and the 2PDA-Au group exhibited the highest metabolic activity, followed by 20PDA-Au (Figure 6B). All three PDA coated groups exhibited significant increases in metabolic activity relative to one another. Similar tendency to increase was evident between 0.2PDA-Au and 2PDA-Au groups (*p* < 0.0001), but 20PDA-Au exhibited a significant decrease in metabolic activity compared to 2PDA-Au (*p* < 0.01), where it was still significantly higher than 0.2PDA-Au (*p* < 0.0001).

The quantitative data were further supported by fluorescence microscopy findings. Relatively limited cell attachment was observed on neat PCL membranes, whereas a greater presence of viable cells was evident on all PDA- and PDA-Au-modified membranes, particularly on day 14 (Figure 6A). The improved cellular response on PDA- and PDA-Au-modified membranes may be attributed to the favorable surface properties conferred by PDA functionalization and the incorporation of AuNPs.

## 4. Discussion

The structural and chemical modifications to the electrospun PCL membranes significantly affected their physicochemical characteristics. The phenomena of surface wettability, swelling kinetics, and density can be thoroughly explained by the synergistic interaction of functional group behavior and the elevated surface energy introduced by the modifications.

Although PCL is considered a promising material in cardiac tissue engineering due to its superior mechanical strength and biocompatibility, its hydrophobic nature impairs cell adhesion [33], and the characteristically dense structure of electrospun networks severely restricts cellular infiltration [34]. In this study, the mean contact angle of 100.05° ± 0.254 measured in the neat electrospun PCL control group perfectly aligns with this classical hydrophobic behavior. To overcome this limitation, PDA modification was applied. Based on a mussel-inspired biomimetic adhesion mechanism, this modification successfully introduced hydrophilic functional groups, such as catechol, amine, and imine, onto the polymer surface [35,36]. Our findings demonstrate that the gradual decrease in water contact angle values, which correlates with coating solution concentration, indicates successful surface functionalization. While no drastic change in surface wettability was observed in the group treated with 0.2PDA (97.24°), the drop in the contact angle to 16.57° in the 20PDA group, with increasing coating solution concentration, indicates that the membrane was successfully modified into a cell-friendly, hydrophilic phase [37,38]. Decorating the modified membranes with AuNPs via in situ reduction produced an exceptional synergistic effect on surface wettability. Although PDA modification alone provided sufficient hydrophilicity, the abrupt decrease in the contact angle to 27.30° upon the addition of AuNPs to the 0.2PDA group (0.2PDA-Au) clearly demonstrates this effect. The abrupt decrease in contact angle upon addition of AuNPs can be attributed to the synergistic interaction between the broad hydrophilic functional groups (catechols and amines) of the PDA network and the exceptionally high surface energy characteristic of AuNPs [39,40].

Another striking finding of our study is that a water droplet placed on the 20PDA-AuNP membrane produced at the highest coating concentration was absorbed by capillary forces within fractions of a second, before a stable spherical boundary could form, thereby exhibiting extreme hydrophilicity. This unique combination of elevated surface energy introduced by AuNPs and the intense chemical hydrophilicity of the high-concentration PDA network, achieved through acidic and alkaline exposure during AuNP decoration, enabled water not only to spread across the surface but also to penetrate the three-dimensional fiber network instantly [41]. The absorption characteristics of both the PDA coating and AuNP incorporation can provide an excellent microenvironment for the homogeneous transport of cellular milieu and culture media (nutrients, oxygen, and growth factors) to the innermost regions of the membrane under both in vitro and in vivo conditions [42,43].

In tissue engineering applications, the swelling capacity and water uptake kinetics of ECM-mimicking scaffolds are critical parameters for facilitating cellular nutrient and oxygen transport, eliminating metabolic waste, and enabling cellular signaling [44]. The water uptake test results obtained in this study indicated that functionalizing electrospun PCL matrices substantially enhances their water absorption capacity, from approximately 435% up to 1290% for PDA coated groups and 1580% for further AuNP decorated groups. This behavior can be attributed to the high surface energy imparted by the metallic nanoparticles on the fiber surface [45], which, together with the hydrophilic PDA network, amplifies capillary forces and facilitates the rapid wicking of liquid into the membrane pores [46]. As highlighted in the literature, mussel-inspired PDA exhibits strong, controlled adhesion to a wide range of materials, forming a stable coating layer [47]. It has also been consistently reported in other recent studies that PDA increases the thickness during the surface modification of hydrophobic fibers, such as PCL and poly(lactic acid), produced by electrospinning [48,49]. This confirms that increasing PDA concentration forms a thicker coating around the polymer network, thereby increasing the composite’s mass and, consequently, its density, increasing with the coating concentration, as indicated by apparent density tests. Incorporation of AuNPs definitely increased density, which can be attributed to the increased mass of dense metallic AuNPs sitting on the fiber surfaces. The gradual decrease in porosity, observed in parallel with the increase in material density, is directly related to the fundamental structural geometry of fiber-based scaffolds. The fact that the highest porosity ratio observed in neat PCL membranes (69%) is largely preserved at low PDA concentrations indicates that the coating at this level does not attain a thickness sufficient to substantially alter the fiber morphology. However, the significant narrowing of inter-fiber void volumes with increasing coating concentration is a physical consequence of the dopamine self-polymerization mechanism. As is well established in the literature, high-concentration PDA applications form an increasingly thick, conformal layer around the nanofibers [36]. When the statistical geometry of two-dimensional random network structures is examined, an increase in fiber width, while maintaining constant network coverage, reduces the fractional open area, thereby decreasing the size of the polygons forming the pores [50].

Evaluation of the electrical characteristics of the fabricated membranes revealed that surface modifications had a profound impact on conductivity. As expected from its polymeric and insulating nature, the pure PCL matrix exhibited the lowest conductivity profile of 3.01 × 10^−8^ ± 2.60 × 10^−9^ S·cm^−1^. The application of PDA onto the fiber surfaces significantly altered this insulating behavior. This PDA-induced enhancement in conductivity is fundamentally attributed to the “electron hopping” mechanism, which facilitates charge transport via the conjugated indole and catechol units within its structure [28]. Increasing the coating concentration elevated the density of these hopping sites; consequently, the conductivity of the 20PDA group reached 8.96 × 10^−6^ ± 6.60 × 10^−6^ S·cm^−1^, demonstrating the formation of a conductive network. While the PDA coating provided a basal conductive platform, the in situ reduction in AuNPs onto this framework exerted a transformative effect, maximizing electron transfer. Structural voids and insulating regions within electrospun polymeric membranes typically impede continuous electron flow. However, the localized integration of AuNPs overcame these physical barriers, enhancing conductivity by functioning as continuous electrical bridges throughout the matrix [51]. The increased concentration of the underlying PDA layer facilitated denser and more homogeneous nucleation of AuNPs. This enabled the AuNPs to reach a percolation threshold and establish a continuous conductive pathway, rather than remaining as isolated islands [52]. As a result of this synergistic interaction, the 20PDA-Au group achieved the highest surface conductivity of 5.85 × 10^−5^ ± 2.38 × 10^−5^ S·cm^−1^. This remarkable electroactivity possesses the potential to successfully mimic the native electrical microenvironment required by excitable cells and assist during the therapy stage.

The two biological tests conducted in this work, extract-based indirect cytotoxicity and direct cytocompatibility assays, evaluate the cytotoxicity of the membranes using both SH-SY5Y cells and primary HCM cells, and include direct long-term culture of primary HCM on the membranes for 7 and 14 days, with Alamar Blue and Live/Dead fluorescence analyses. The main purpose of the extract-based indirect MTT study was to determine the potential toxicity of the chemicals that leaked from the membranes. Instead, the Alamar Blue experiment assessed the metabolic activity and cytocompatibility of primary HCMs upon direct contact with the membranes. These two tests complement each other and show how the changed surface physicochemical properties of the material affect the ability of cells to survive over time [53]. In vitro cytotoxicity assessment studies revealed that modifying PCL membranes with PDA and in situ reducing AuNPs caused a slight, concentration-dependent decrease in cell viability relative to the unmodified PCL control group after 24 h of incubation. The neat PCL membranes exhibited higher cell viability for both cell types within the first 24 h. Yu et al. (2022) [54] demonstrated that while PDA coatings enhance cell adhesion, morphological and kinetic adaptation processes during the initial cell attachment to the modified surface may lead to significant decreases in cell viability measurements in the first few hours. However, this behavior is not due to the intrinsic cytotoxicity of PDA [54]. It should be noted that SH-SY5Y neuroblastoma cells were included in this evaluation not to claim a neural tissue engineering application, but rather to serve as a complementary, highly sensitive human cell model [55]. Their inclusion aimed to assess whether potential leachable components (such as unreacted dopamine or AuNP residues) from the modified membranes induced toxicity in a second human cell line, in addition to primary HCM [56]. Cell viability results after a 48 h cultivation period show that both HCM and SH-SY5Y cells are adapted and stabilized, but HCM cells exhibited a better adaptation than SH-SY5Y cells to this specialized environment. All PDA-Au groups in both cell types showed lower viability than their PDA-coated counterparts. It has been reported that the initial interaction between cells and AuNPs may promote the generation of reactive oxygen species (ROS), which may temporarily inhibit mitochondrial function and cellular metabolism [57]. The cytotoxic profile of AuNPs is determined by factors such as concentration, size, surface charge, and roughness, thereby regulating apoptosis or necrosis in cells. The AuNPs deposited on the PDA surface by the in situ reduction method used in this study did not reach the toxic threshold that would trigger significant necrotic damage; instead, they caused only a slight 24 h metabolic slowdown during the cells’ adaptation to the newly created nanostructured surface.

The neat PCL membranes were naturally hydrophobic, resulting in poor initial cell attachment to the matrix and the lowest metabolic activity among all groups. This observation is consistent with the findings of Ghasemi-Mobarakeh et al. (2008), who reported that although PCL-based scaffolds exhibit superior mechanical strength and biocompatibility, their hydrophobic nature significantly reduces cell adhesion [33]. Conversely, surface modification using PDA promoted metabolic activity in a concentration-dependent manner, with a significant effect by day 14; the 20PDA group exhibited a more pronounced cellular response than the neat PCL control. This cellular enhancement is primarily the result of a large number of active functional groups (catechols and amines) attached to the surface via PDA functionalization, and of the substantial decrease in the water contact angle, both of which create a biologically favorable substrate for cell attachment. Ku et al. (2010) proposed PDA as a “general functionalization route for cell adhesion on non-wetting surfaces” and demonstrated that depositing a PDA ad-layer onto polymeric surfaces can significantly enhance endothelial and myocardial cell attachment [35]. It indicates that non-wetting surfaces are inherently limited in their ability to support cell attachment. Zhang et al. (2024) also noted that the various functional groups in the PDA coating structure can form broad noncovalent interactions with endogenous ECM proteins secreted by cells, thereby providing an ideal framework for long-term cell proliferation [58].

One of the most notable results of this study is that in situ incorporation of AuNPs further enhanced cellular activity, culminating in the highest metabolic activity capacity by day 14 (2PDA-Au). This was confirmed by fluorescence microscopy of cells cultured on surfaces with increased PDA and AuNP modification levels, which exhibited a clearly elongated morphology typical of mature cardiac myocytes, with well-developed cytoskeletal extensions and spreading strongly over the substrate [23]. As stated in the relevant literature, “gold nanoparticle-integrated scaffolds for tissue engineering and regenerative medicine” are highly cytocompatible nanostructures that preserve the electrical integrity of cardiac tissues and strongly improve the cardiac cell proliferation [59] for the synergistic interplay in which the high surface affinity and elevated surface energy induced by AuNP loading on the hydrophilic PDA layer optimize the cellular proliferation [60]. In summary, the synergy between biomimetic adhesion, increased hydrophilicity, and the high surface energy of localized AuNPs creates a highly favorable microenvironment for attachment, morphological spreading, and long-term proliferation of primary cardiac cells in the membrane matrix.

## 5. Conclusions

This study demonstrates the successful fabrication of PDA- and AuNP-modified electrospun PCL fibrous membranes with potential for use as cardiac patches. The synergistic integration of PDA and AuNPs transformed the inherently hydrophobic PCL into an extremely hydrophilic microenvironment, significantly increasing electrical conductivity by approximately 1000-fold (up to 5.85 × 10^−5^ S·cm^−1^) without compromising structural properties. In vitro cytotoxicity assays confirmed that the electrospun PCL membranes with enhanced electrical conductivity are non-toxic to SH-SY5Y and HCM cells. Direct cytocompatibility assessment revealed that they support HCM cell proliferation and metabolic activity. It has been concluded that the membranes produced in this study have potential for use as cardiac patches. Future in vivo studies using a suitable animal model are to evaluate the efficacy and therapeutic potential of these structures for use in therapy after MI. Furthermore, advanced functional assessments, including confocal immunofluorescence for α-actinin and Cx43 to verify cardiomyocyte structural organization, Ca^2+^ transient imaging, and the evaluation of antibacterial properties, will form the foundation of our upcoming in vivo and advanced in vitro studies.

## Figures and Tables

**Table 1:** Diameters of the electrospun fibers and AuNPs calculated using SEM images.

Group	Fiber Diameter (µm) (Mean ± Std)	AuNP (nm) (Mean ± Std)
PCL	2.44 ± 0.52	-
0.2PDA	2.49 ± 0.38	-
2PDA	2.62 ± 0.17	-
20PDA	2.91 ± 0.31	-
0.2PDA-Au	2.48 ± 0.27	41 ± 14 *
2PDA-Au	2.64 ± 0.32	63 ± 27 *
20PDA-Au	2.74 ± 0.29	88 ± 31 *

*: Statistically significant difference (*p* < 0.05).

**Table 2:** Characterization data of the neat, PDA-, and PDA-Au coated PCL fibers.

Group	Density (g·cm^−3^) (Mean ± Std)	Porosity (%) (Mean ± Std)	Contact Angle (°) (Mean ± Std)	Water-Uptake (%) (Mean ± Std)
PCL	0.35 ± 0.03	69.14 ± 2.96	100.05 ± 0.25	435.5 ± 28.7
0.2PDA	0.36 ± 0.03	68.49 ± 2.29	97.24 ± 4.75	462.5 ± 19.0
2PDA	0.43 ± 0.02	62.24 ± 1.62	57.42 ± 1.91	1032.3 ± 69.4
20PDA	0.47 ± 0.01	58.98 ± 1.29	16.57 ± 0.51	1290.9 ± 142.9
0.2PDA-Au	0.37 ± 0.02	67.34 ± 1.55	27.30 ± 0.87	1008.4 ± 97.6
2PDA-Au	0.45 ± 0.03	60.81 ± 2.99	18.30 ± 1.79	1318.1 ± 49.4
20PDA-Au	0.48 ± 0.04	58.00 ± 3.22	N/A *	1584.0 ± 127.7

*: Contact angle measurements were not possible due to sudden droplet disappearance at measurement timepoints.

**Table 3:** Mechanical properties of the electrospun PCL, PDA-modified PCL, and Au-decorated PDA-PCL fibers.

Group	UTS (MPa) (Mean ± Std)	Fracture Strain (mm·mm^−1^) (Mean ± Std)	Elastic Modulus (MPa) (Mean ± Std)
PCL	7.42 ± 1.60	4.68 ± 1.51	41.71 ± 5.63
0.2PDA	7.38 ± 1.72	4.71 ± 1.54	42.44 ± 6.71
2PDA	8.18 ± 1.84	4.52 ± 1.43	45.19 ± 7.13
20PDA	8.55 ± 1.87	4.17 ± 1.42	46.32 ± 7.09
0.2PDA-Au	7.52 ± 1.71	4.59 ± 1.45	41.95 ± 6.97
2PDA-Au	7.64 ± 1.76	4.78 ± 1.51	44.05 ± 7.22
20PDA-Au	8.02 ± 1.79	4.10 ± 1.39	47.65 ± 7.46

**Table 4:** Surface electrical conductivity of the PCL and PDA/Au modified membranes.

Group	σ (S·cm^−1^) (Mean ± Std)
PCL	3.01 × 10^−8^ ± 2.60 × 10^−9^
0.2PDA	5.87 × 10^−7^ ± 1.42 × 10^−7^
2PDA	5.53 × 10^−7^ ± 1.82 × 10^−7^
20PDA	8.96 × 10^−6^ ± 6.60 × 10^−6^
0.2PDA-Au	1.16 × 10^−6^ ± 3.03 × 10^−7^
2PDA-Au	1.77 × 10^−5^ ± 3.78 × 10^−7^
20PDA-Au	5.85 × 10^−5^ ± 2.38 × 10^−5^

## Data Availability

The original contributions presented in this study are included in the article. Further inquiries can be directed to the corresponding author.

## Supplementary Materials

The following supporting information can be downloaded at: https://www.mdpi.com/article/10.3390/polym18192408/s1; Figure S1. Representative stress (MPa) − strain (mm/mm) curves for the test groups.

## Abbreviations

The following abbreviations are used in this manuscript:

MI	Myocardial Infarction
ECM	Extracellular Matrix
PCL	Polycaprolactone
PDA	Polydopamine
AuNP	Gold Nanoparticle
HCM	Human Cardiac Myocyte
SEM	Scanning Electron Microscopy
