# Supplementary material for: Dual-Functionalized Electrospun PCL Membranes with Polydopamine/Gold Nanoparticles: Cytocompatibility Assessment for a Potential Cardiac Patch
Source: Polymers (Basel). 2026 Oct 2;18(19):2408. doi: 10.3390/polym18192408 (PMC13666718; doi:10.3390/polym18192408)
Supplement: Supplementary file 1 [file polymers-18-02408-s001.zip › polymers-4547534-supplementary.pdf]

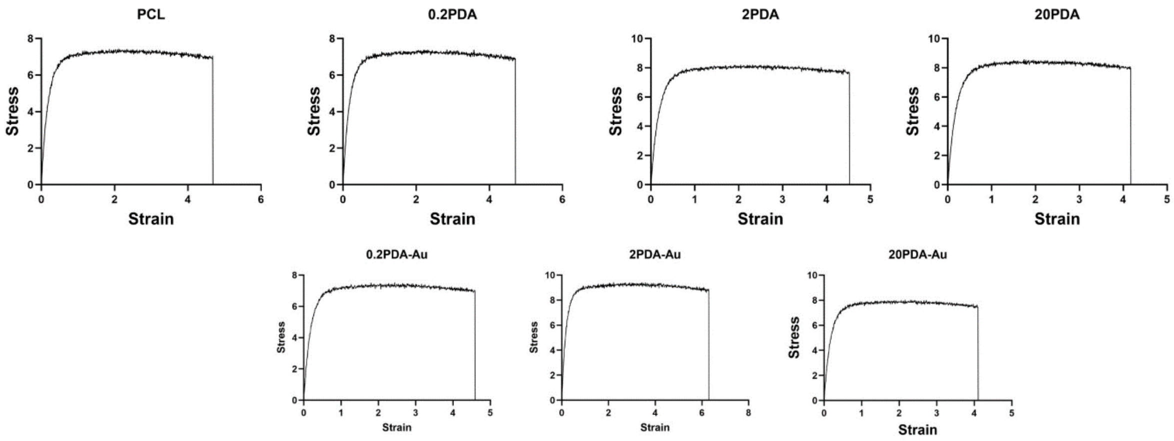

Figure S1. Representative stress (MPa) – strain (mm/mm) curves for the test groups.
